# Two-to-one color-response mapping and the presence of semantic conflict in the Stroop task

**DOI:** 10.3389/fpsyg.2014.01157

**Published:** 2014-10-14

**Authors:** Nabil Hasshim, Benjamin A. Parris

**Affiliations:** Department of Psychology, Faculty of Science and Technology, Bournemouth UniversityPoole, UK

**Keywords:** Stroop, semantic, conflict, same-response, non-response

## Abstract

A series of recent studies have utilized the two-to-one mapping paradigm in the Stroop task. In this paradigm, the word red might be presented in blue when both red and blue share the same-response key (same-response trials). This manipulation has been used to show the separate contributions of (within) semantic category conflict and response conflict to Stroop interference. Such results evidencing semantic category conflict are incompatible with models of the Stroop task that are based on response conflict only. However, the nature of same-response trials is unclear since they are also likely to involve response facilitation given that both dimensions of the stimulus provide evidence toward the same-response. In this study we explored this possibility by comparing them with three other trial types. We report strong (Bayesian) evidence for no statistical difference between same-response and non-color word neutral trials, faster responses to same-response trials than to non-response set incongruent trials, and no differences between same-response vs. congruent trials when contingency is controlled. Our results suggest that same-response trials are not different from neutral trials indicating that they cannot be used reliably to determine the presence or absence of semantic category conflict. In light of these results, the interpretation of a series of recent studies might have to be reassessed.

## INTRODUCTION

The classic Stroop task (Stroop, 1935) requires participants to respond as quickly and as accurately as possible to the color in which a word is printed whilst ignoring the word’s meaning. The *Stroop congruency effect* refers to the slower response times (RTs) on incongruent trials (e.g., the word “red” printed in blue) compared to congruent trials (e.g., the word “red” printed in red). This effect has been attributed to having to resolve conflict at the response stage when the color and the meaning of the word each activate different-responses (referred to as response conflict or stimulus-response conflict, Cohen et al., 1990; MacLeod, 1991; Roelofs, 2003). However, some researchers have posited that in addition to interference/conflict resolution at the response stage, performance in the Stroop task also requires conflict resolution in earlier processing stages (e.g., Klein, 1964; Sharma and McKenna, 1998; Zhang and Kornblum, 1998; Zhang et al., 1999; De Houwer, 2003; Schmidt and Cheesman, 2005). For example, semantic category conflict (an example of stimulus-stimulus conflict, or conflict that arises during stimulus processing independently of response processes) refers to when both dimensions of the stimulus elicit two different items from the same semantic category and thus produce within-category competition. In the case of a typical Stroop task, both the word and color dimensions activate color concepts which results in competition at the semantic category level of “colors”. It should be noted that studies in the literature typically use the general term “semantic conflict” while the current research defines semantic category conflict as its main source.

In an effort to distinguish response conflict and semantic category conflict researchers (De Houwer, 2003; Schmidt and Cheesman, 2005; van Veen and Carter, 2005; Steinhauser and Hubner, 2009) have used a variation of the Stroop task first introduced in De Houwer (2003) that maps two color responses to one response button. Typically in studies employing the Stroop task, each response is assigned to a particular key on the keyboard or response box. This ensures that when an incongruent word is presented (e.g., “red” in blue) the font color and word will contribute evidence toward *different*-response keys (i.e., “red” will be assigned to the “z” on the keyboard and “blue” will be assigned to the “m” key), ensuring competition at the response output level. It is possible, however, to assign both “red” and “blue” to the “z” key. When the incongruent word *red* is presented in blue both dimensions of the Stroop stimulus contribute evidence toward the *same*-response keys, but still activate different color concepts. This two-to-one paradigm enables a distinction between two types of incongruent trials determined by whether the relevant and irrelevant stimuli share a common response. We will refer to these incongruent trials as *different-response* and *same-response trials*, respectively. Same-response trials are thought to involve semantic category conflict but not response conflict (since both “red” and “blue” share a common response) while different-response trials involve both semantic and response conflict.

This paradigm has been used to differentiate semantic and response based conflict. Comparing different-response trials to same-response trials is thought to yield a pure measure of response conflict, while comparing same-response trials to congruent trials is thought to measure semantic category (or sometimes called stimulus-stimulus) conflict (De Houwer, 2003; Schmidt and Cheesman, 2005; van Veen and Carter, 2005; Steinhauser and Hubner, 2009). Since congruent trials are also trials on which both dimensions of the stimulus contribute evidence toward the same-response, but also contribute evidence toward the same semantic item, it is assumed that the difference between the two conditions is semantic category conflict. In short, same-response trials are semantic category-incompatible but response-compatible, different-response trials are both semantic category-incompatible and response-incompatible and congruent trials are both semantic category compatible and response- compatible.

Schmidt and Cheesman (2005) observed a 24 ms semantic category conflict effect and a 32 ms response conflict effect. In an fMRI study, van Veen and Carter (2005) compared brain activity associated with response and semantic conflict and showed that each activated unique brain areas. They found that the contrast between same-response and congruent trials, reflecting semantic category conflict, did not overlap with the contrast between different-response and same-response trials. This was taken as evidence for the two types of conflict being detected and resolved by distinct regions of the brain. Using ex-Gaussian distribution analysis, Steinhauser and Hubner (2009) used same-response trials to get a purer measure of response conflict and observed response conflict in the Gaussian component of the distribution while task conflict (a form of semantic based conflict) was observed in the exponential component. Highlighting its utility, other recent studies have also employed the paradigm or similar two-to-one mapping paradigms (Wendt et al., 2007; Chen et al., 2011, 2013; Berggren and Derakshan, 2014).

In sum, in the present literature there is a debate as to whether semantic processes contribute to Stroop effects. Same-response trials have been used to provide evidence for the influences of semantic processes in the Stroop task, particularly semantic category conflict. According to some models such conflict should not exist since according to these models all interference in Stroop-like tasks is attributable to response conflict (Cohen et al., 1990; Roelofs, 2003). In light of the uptake of this paradigm, and the theoretical ramifications of the presence of semantic category conflict, the present study sought to assess whether one can measure the contribution of semantic category conflict to Stroop effects using same-response trials. In Experiment 1 we aimed to replicate the semantic category conflict effect observed in previous studies. In Experiment 2, participants completed two counterbalanced blocks of the Stroop task. In one block, consistent with previous studies and Experiment 1, participants were exposed to congruent, same-response and different-response trials. In this block, non-color word neutral trials (e.g., “stage” in blue) were also included. In the other block, the congruent stimuli were replaced with non-response set incongruent stimuli (i.e., stimuli in which the word dimension is a color word that is not one of the possible response colors, e.g., “purple” in red). Furthermore, in both blocks we controlled for response contingency (Schmidt et al., 2007; Schmidt and Besner, 2008). We explain the motivation for each of these modifications below.

### INCLUSION OF NON-COLOR WORD NEUTRAL TRIALS

There is a potential issue with calculating semantic category conflict by comparing same-response trials to congruent trials as all previous studies have done. This is because, whilst congruent and same-response trials could involve response facilitation because the color concepts from both dimensions in each case provide evidence toward the same-response, congruent trials likely involve a unique semantic facilitation effect (Brown, 2011) which would result in faster RTs. Thus, this might not make them a suitable baseline to isolate semantic conflict since any difference in RT between the two trial types could be due in part to the presence of semantic facilitation. In order to remove the influence of semantic facilitation, Experiment 2A included non-color word neutral trials which do not involve semantic or response facilitation or semantic or response conflict. Slower RTs on same-response trials compared to neutral trials would be supportive evidence of semantic category conflict, as is predicted by multiple-stage accounts (Klein, 1964; Zhang and Kornblum, 1998; De Houwer, 2003; Schmidt and Cheesman, 2005; Zhang et al., 1999). Should same-response trials be faster than neutral trials it would be evidence for an effect of response facilitation on same-response trials, not solely semantic conflict as has previously been assumed. Moreover, it would mean that studies comparing same-response and different-response trials for a purer measure of response conflict would also have to be reassessed. Importantly, even evidence for no difference between the trial types would be meaningful since it would indicate that same-response trials should not be used to infer the presence or absence of semantic category (or stimulus–stimulus) conflict.

### INCLUSION OF NON-RESPONSE SET INCONGRUENT TRIALS

Non-response set incongruent trials (e.g., “purple” printed in blue, when the color purple is not used on any trial) involve semantic category competition but no semantic facilitation, since both dimensions of the Stroop stimulus activate different color concepts, but little or no response competition (Klein, 1964; Sugg and McDonald, 1994) and response facilitation because the word dimension is not a possible response. If responses to same-response trials are faster than those to non-response set trials it would provide support for the existence of response facilitation on the former. Moreover, since non-response set trials do not include response facilitation, the comparison between these trials and neutral trials might give a better measure of semantic category conflict than same-response trials. Finally, the comparison between non-response set trials and different-response trials might provide a purer measure of response competition.

### CONTROLLING FOR RESPONSE CONTINGENCY

Recent work has shown effects of contingency on congruent trial RTs (Schmidt et al., 2007; Schmidt and Besner, 2008). The contingency effect shows that the associations between word and response are implicitly learnt throughout an experiment and used to predict specific responses to each word, which facilitates RTs to trials where the correct response is highly correlated to the word. This is the case with congruent trials since they often make up half the trials. For example, with a four response Stroop task there are only four possible word-color combinations to create the congruent stimuli whereas there are a possible 12 word-color combinations when creating incongruent stimuli. This means that the words are more often associated with their congruent color counterparts. When contingency is absent, RTs to congruent trials increase (see Schmidt et al., 2007; Schmidt and Besner, 2008). Although not explicit, contingency has been controlled in some studies employing same-response trials (De Houwer, 2003; Schmidt and Cheesman, 2005), whilst it was not controlled in others (van Veen and Carter, 2005; Steinhauser and Hubner, 2009). Importantly for present purposes, contingency is also likely to affect same-response trials. Since Experiment 2A involved congruent trials, we controlled for contingency by having twice as many different-response trials than congruent and same-response trials, which ensures that for each color word, the probability of any of the responses being the correct response is be equal. Thus, any difference remaining between same-response/congruent trials and other trials types would therefore represent influences attributable to other factors.

### SUMMARY

Thus the main goal of the current research was to determine whether same-response trials truly index semantic category conflict by addressing possible influences of semantic and response facilitation whilst controlling for contingency. The critical comparisons in the experiment were as follows: (1) Same-response trials vs. neutral trials: the difference between these trials would be a more accurate measure of semantic category competition since neutral trials involve neither response facilitation nor semantic category conflict; (2) Same-response trials vs. non-response set trials: the comparison of these trials would also inform us whether there is facilitation involved when processing the former as an inhibition only based account of same-response trials predicts no difference between the two, while one that includes a response facilitation component would predict faster responses to same-response trials; (3) Same-response trials vs. congruent trials when contingency is controlled: If contingency does have an effect, we would expect the difference between the two conditions to be smaller when it has been controlled for; (4) Same-response trials vs. different-response trials when contingency is controlled: If contingency is affecting RTs to same-response trials the difference observed between these two trial types in some previous studies is likely to overestimate response competition.

Before reporting the key experiment of the paper (Experiment 2), we first report a replication (Experiment 1) of the two-to-one mapping paradigm as it has been most commonly employed: Including different-response, same-response and congruent trials but without neutral and non-response set trials and without controlling for contingency. To foreshadow the findings of this paper, using Bayesian statistics we provide evidence for no difference between neutral and same-response trials suggesting that studies utilizing same-response trials to measure semantic category conflict or response conflict will have to be reassessed.

Experiment 1 is reported to establish the magnitude of the effects under present conditions and for later use in the calculation of Bayes Factors where we test whether any null effects observed are evidence for the absence of an effect or the absence of evidence for an effect and was not run as a within-subjects manipulation with Experiment 2 to avoid learned contingencies carrying over. Experiment 2 consisted of two counterbalanced blocks of trials in which contingency was controlled. In one block, only neutral, same-response, congruent and different-response trials were included (Experiment 2A). The other block was the same except that the congruent trials were replaced by non-response set trials (Experiment 2B).

## MATERIALS AND METHODS

### PARTICIPANTS

Two different groups of 36 students (12 male in Experiment 1, 6 in Experiment 2) participated in each of the experiments in exchange for course credit or £5. The average age was 24.7 (SD = 6.4) for Experiment 1 and 21.0 (SD = 5.0) for Experiment 2.

### APPARATUS AND MATERIALS

Stimuli were presented using standard PC running Experiment Builder software (SR Research Ltd, 2010) and responses were made via a standard chiclet keyboard with colored stickers on the corresponding response keys. In Experiment 1, the colors blue (RGB: 0; 112; 192) and green (RGB: 0; 255; 0) were assigned “c” key while red (RGB: 255; 0; 0) and yellow (RGB: 255; 255; 0) the “m” key.

For Experiment 2 the neutral words used were DUE, WALL, STORY, and MARVEL. In addition to the colors used in Experiment 1, the colors orange (RGB: 255; 127; 0), pink (RGB: 255; 20; 147), purple (RGB: 0; 125; 255), and white (RGB: 255; 255; 255) were used. For each participant, four of the colors were used as responses while the other four were used as the word dimension in the non-response trials. The colors that were assigned as responses and distractors were counterbalanced as was which colors were mapped on to the response keys and the order of which participants performed Experiments 2A,B. Words in each condition had been matched for frequency and length using the English Lexicon Project (Balota et al., 2007). Each word was presented in the four response colors equally often. The words were presented in lowercase, bold, and in size 20 Courier New font on a black background.

### PROCEDURE

On each trial, participants were presented with a gray fixation cross in the center of the screen for 500 ms followed by the Stroop stimulus which remained on the screen until a response was made. They were instructed to press the assigned key corresponding to the color of the text as quickly as possible whilst ignoring the meaning of the word. An auditory feedback tone was given when an error was made. Participants went through a practice block of 48 trials. Before the experiment participants were given instructions verbally and written instructions were presented on the screen before each block commenced.

In Experiment 1, participants went through four blocks of 72 trials, resulting in 96 experimental trials in each condition in total. Each block contained an equal number of trials from the three conditions (congruent, same-response, and different-response) presented in random order.

In Experiment 2A, participants went through three blocks of 80 trials, which consisted of 48 trials each of the congruent, same-response, and neutral conditions and 96 trials of the different-response condition. Having twice as many different-response trials is necessary to control for contingency by ensuring that the correct response to each word presented is equal for the two response buttons.

In Experiment 2B, participants went through three blocks of 64 trials which consisted of 48 trials each of the same-response, different-response, neutral, and non-response trials. It was not necessary to have different number of trials of each trial type as congruent trials were not presented.

## RESULTS

### EXPERIMENT 1

Incorrect responses (5.2% across all conditions) were excluded from the analyses along with responses that were faster than 200 ms and slower than 2500 ms. This resulted in the total proportion of valid responses to be 94.6%.

**FIGURE 1:**
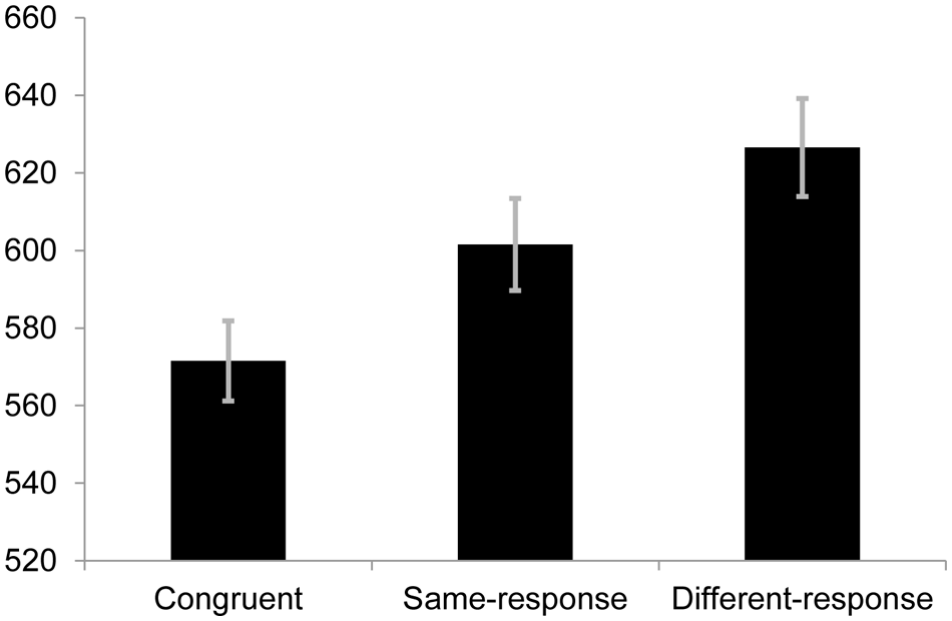
**Mean response times (RTs; in ms) for each condition in Experiment 1.** Error bars represent SE.

We conducted a one-way repeated measures analysis of variance (ANOVA) to determine whether there were differences in RTs to the congruent, same-response and different-response conditions. The difference across the three groups was significant [*F*(2,70) = 31.32, *p* < 0.001, *r* = 0.56]. *A priori* follow-up tests revealed that RTs for the congruent condition (*M* = 571.53 ms, SE = 20.68) were significantly faster than those on same-response trials [*M* = 601.56 ms, SE = 23.69; *t*(35) = 4.42, *p* < 0.001, *r* = 0.60] and different-response trials [*M* = 626.54 ms, SE = 25.34; *t*(35) = 7.15, *p* < 0.001, *r* = 0.77] while the same-response condition was faster than the different-response condition [*t*(35) = 3.95, *p* < 0.001, *r* = 0.56]. Importantly, these results replicate the findings from previous studies showing a semantic category conflict effect (see **Figure 1**).

The omnibus ANOVA for error rates across the three conditions was statistically significant [*F*(2,70) = 12.85, *p* < 0.001, *r* = 0.39]. Follow-up pairwise comparisons showed that the error rate in the different condition (6.8%) was significantly more than the same-response [4.4%; *t*(35) = 3.87, *p* < 0.001, *r* = 0.54] and congruent [4.5%; *t*(35) = 4.03, *p* < 0.001, *r* = 0.56] conditions. The error rates between same-response and congruent trials were non-significantly different [*t*(35) = 0.378, *p* = 0.708, *r* = 0.06].

### EXPERIMENT 2A

**FIGURE 2:**
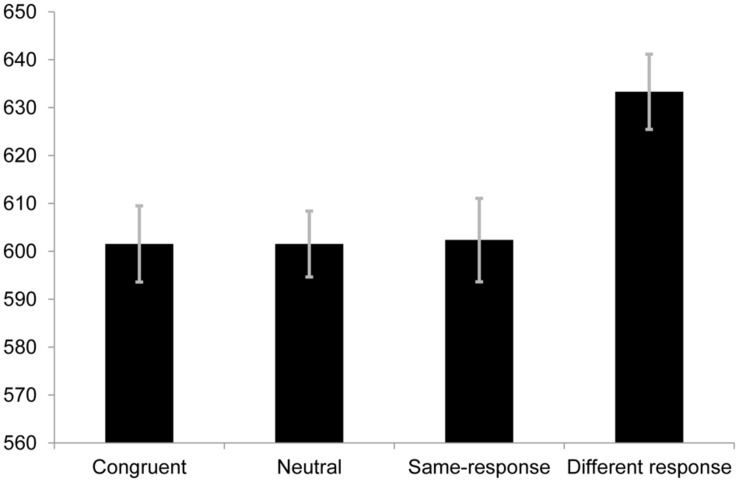
**Mean RTs (in ms) for each condition in Experiment 2A.** Error bars represent SE.

The same exclusion criteria as Experiment 1 were used which resulted in the proportion of valid responses to be 95.5%. A one-way repeated measures ANOVA was conducted and was found to be statistically significant [*F*(3,105) = 8.72, *p* < 0.001, *r* = 0.23; see **Figure 2**]. In the introduction a set of critical comparisons were outlined. Data from this block permit us to test critical comparisons 1, 3, and 4.

No difference was observed between same-response (*M* = 602.35 ms, SE = 17.40) and neutral (*M* = 601.55 ms, SE = 13.75) trials [*t*(35) = 0.089, *p* = 0.929, *r* = 0.015]. To determine if there was evidence for no difference between the two conditions, we used a Bayes Factor (Dienes, 2011), where we contrasted the theory that there was a difference between the two conditions with the null hypothesis that there was no difference (0.33 and below being the cut off for strong evidence for the null; a Bayes Factor of 3 or above can be taken as strong evidence for a difference). To calculate the Bayes Factor we used 6–45 ms as the range and assumed a uniform distribution (i.e., all values within this range were equally likely). This range was chosen based on previous work in our and other labs (De Houwer, 2003; Schmidt and Cheesman, 2005; van Veen and Carter, 2005; Chen et al., 2011, 2013; Parris et al., 2012a,b, 2013; Parris and Dienes, 2013) considering the theory under test (i.e., that semantic category conflict exists/is measurable using same-response trials). ^1^ For the difference between neutral and same-response trials a Bayes Factors of 0.17 was returned, providing strong evidence for the null hypothesis of no difference relative to the alternative hypothesis. In other words the observed mean difference and SE of the difference between the same-response and neutral trials were sufficiently far from the expected range to be considered evidence for the null. This finding is important and suggests that, at least when using RT as the dependent variable, same-response trials do not index semantic category competition.

For critical comparison 3 we calculated a Bayes Factor for the difference between congruent (*M* = 601.54 ms, SE = 15.90) and same-response (*M* = 602.35 ms, SE = 17.40) trials [*t*(35) = 0.095, *p* = 0.925, *r* = 0.016]. Again we assumed a uniform distribution with all values between 6 and 45 ms being equally likely. This yielded a Bayes Factor of 0.15 providing strong evidence for no difference between the two conditions. This finding contrasts with previous studies showing a semantic category conflict effect when contingency is controlled (De Houwer, 2003; Schmidt and Cheesman, 2005).

For critical comparison 4 we compared same-response (*M* = 602.35 ms, SE = 17.40) trials and different-response (*M* = 633.31 ms, SE = 15.7) trials when contingency was controlled. As in Experiment 1 here we observed a significant difference between the two conditions [*t*(35) = 4.54, *p* < 0.001, *r* = 0.61].

Although not one of the stated critical comparisons the large apparent effect of contingency on congruent trial RTs was surprising enough to motivate a comparison between the congruent and neutral trials. It was stated that faster RTs on congruent vs. neutral trials would be attributed to facilitation that remains after contingency is controlled, but there was no statistical difference between the congruent and neutral trial RTs (*p* > 0.05) in this study. We modeled the predictions of the theory of a difference with a uniform between 0 and 30 ms, i.e., any effect was as plausible as any other in the full range (encompassing the 15–27 ms range suggested by the previous work alluded to above). The difference between the congruent (*M* = 601.54 ms, SE = 15.90) and neutral (*M* = 601.55 ms, SE = 13.75) conditions showed a Bayes Factor of 0.29. This result suggests that once contingency is controlled there remains no facilitation effect when using a non-word neutral trial as the baseline. As far as we are aware, this is the first report of this finding, and one that suggests that debates over the mechanisms behind facilitation (MacLeod and MacDonald, 2000; Kane and Engle, 2003; Brown, 2011; Roelofs, 2010) should first consider contingency.

Importantly however, this result also serves another purpose, helping us to interpret the null difference between same-response and congruent trials. This will be discussed later.

The error rates for the congruent, neutral, same-response and different-response were 4.6, 4.3, 3.2, and 4.7% respectively. Analysis of the error rates showed a non-significant difference in the omnibus one-way ANOVA [*F*(3,105) = 2.40, *p* = 0.072, *r* = 0.15].

### EXPERIMENT 2B

**FIGURE 3:**
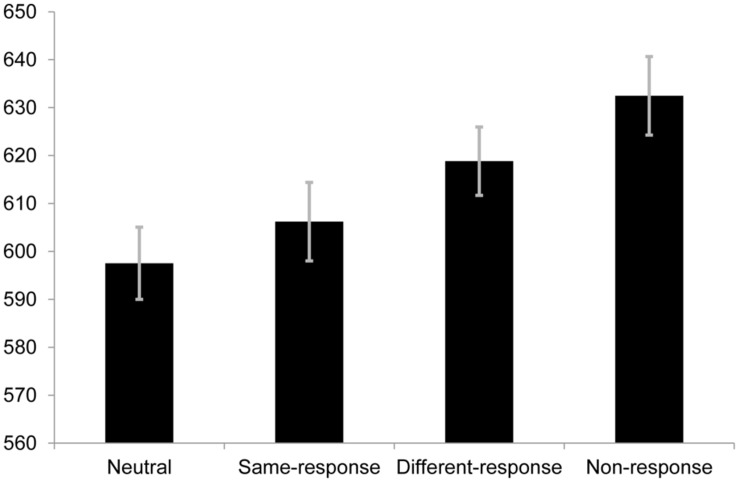
**Mean RTs (in ms) for each condition in Experiment 2B.** Error bars represent SE.

Using the same exclusion criteria as the other two experiments, the proportion of valid responses in this experiment was 94.47%. The repeated measures one-way ANOVA containing all four conditions was statistically significant [*F*(3,105) = 7.71, *p* < 0.001, *r* = 0.26; see **Figure 3**]. To test critical comparison 2, a pairwise comparison was made between the RTs of same-response (*M* = 606.21 ms, SE = 16.36) and non-response set (*M* = 632.48ms, SE = 16.39) trials. The difference was statistically significant [*t*(35) = 3.49, *p* = 0.001, *r* = 0.51]. This indicated that the non-response set condition had slower RTs than the same-response condition and is supportive of the notion that same-response trials involve response facilitation. However, the pattern of RTs observed encouraged the comparison of the different-response (*M* = 618.83 ms, SE = 14.25) and non-response set trials; a comparison which yielded [*t*(35) = 1.74, *p* = 0.091, *r* = 0.28]. Slower (but statistically non-significant) RTs to non-response set trials compared to different-response trials was unexpected and makes the difference between same-response and non-response trials difficult to interpret.

Since neutral and same-response trials were used in this block, we compared RTs to these trials to see if the same pattern of results from critical comparison 1 of Experiment 2A would be replicated. Using the same criteria employed to calculate the Bayes Factor in Experiment 2A, the non-significant [*t*(35) = 1.07, *p* = 0.294, *r* = 0.18] difference between the two conditions returned a Bayes Factor of 0.58 a value that cannot be taken as evidence for nor against the theory under test (Dienes, 2011) and is therefore not considered further.

The error rates for the neutral, same-response, different-response and non-response trials were 5.8, 4.7, 7.2 and 3.9% respectively. Analysis of the error rates showed a significant difference in the omnibus one-way ANOVA [*F*(3,105) = 3.40, *p* = 0.021, *r* = 0.18]. *Post hoc* pairwise comparisons between the conditions yielded a significant difference between different-response and non-response trials [*t*(35) = 3.31, *p* = 0.012, *r* = 0.49] while the other comparisons were non-significant (*p*s > 0.05). The error rate for different-response trials in the present experiment is much higher than in Experiment 2A [*t*(35) = 2.03, *p* = 0.050, *r* = 0.33], but was only statistically different from the non-response set trials which is largely consistent with the previous block in that errors were no different between different-response, same-response and neutral trials. This is discussed further below.

It is also possible that the introduction of non-response trials influence participants’ approach to different-response trials in Experiment 2B since the number of incongruent trials increases. Pairwise comparisons between the RTs and error rates of different-response trials in the two experiments were run. The results were inconclusive as although the error rates in Experiment 2B were higher the RTs were non-significantly different [*t*(35) = 1.56, *p* = 0.125, *r* = 0.25].

## DISCUSSION

The goal of the present study was to assess the utility of the two-to-one mapping manipulation and the nature of same-response incongruent trials in the Stroop task. This was assessed by comparing them to non-color word neutral trials and non-response set trials whilst controlling for response contingency. The key result is the finding of strong (Bayesian) evidence for no statistical difference between same-response and non-color word neutral trials. As stated earlier, two possible scenarios could be the cause of this: either same-response trials involve both response facilitation and semantic category competition, with the two effects canceling each other out, or the more parsimonious explanation that same-response trials do not involve either effect. Although this result does not allow us to draw conclusions about the mechanisms involved in same-response trials, it shows clearly that same-response trials do not permit a reliable measure of the presence or absence of semantic category conflict and therefore all future studies using the 2-to-1 mapping paradigm should include a neutral baseline.

Same-response incongruent trials were also compared to non-response set trials. Following the assumptions of the two-to-one paradigm, these trials are thought to involve semantic category conflict and not response conflict, just like same-response trials, but in contrast to same-response trials are unlikely to involve response facilitation. We found that non-response set trials were responded to more slowly than same-response trials. This result suggests that RTs to same-response trials are at least partially determined by response facilitation. In light of these results, the significance of a series of recent studies might have to be reassessed (Schmidt and Cheesman, 2005; van Veen and Carter, 2005; Wendt et al., 2007; Steinhauser and Hubner, 2009; Chen et al., 2011, 2013; Berggren and Derakshan, 2014).

However, the longer RT to non-response set trials has to be interpreted with caution since we also observed unexpected results when comparing non-response to different-response trials. RTs to non-response set trials were not different from those to different-response trials, which was not in line with predictions based on previous research. However, recent work in our lab shows that the putative response set effect (different-response trials – non-response set trials) is strongly modulated by trial type mixing and is thus not as reliable as previously thought. Hasshim and Parris (submitted) have shown that the response set effect is much larger when different-response and non-response set trials are presented in different, pure blocks. When presented in mixed blocks the response set effect was substantially reduced; an effect that resulted from a substantial decrease in RT to different-response trials, whilst no other trial type was affected. Thus, since the present results mirror effects observed in Hasshim and Parris, it is likely that trial type mixing employed here is responsible for the lack of the expected response set effect. Moreover, this means that the RTs observed to the non-response set trials are reliable. Indeed a few studies have reported no difference between non-response and different-response trials under similar mixed conditions (but slightly different presentation formats; e.g., Stirling, 1979; Sugg and McDonald, 1994; Milham et al., 2001). However, the error data from Experiment 2B bear consideration at this point. Whilst the number of errors did not differ from those in the neutral or same-response condition, there were significantly fewer errors in the non-response set condition than in the different-response condition. Assuming that the error trials are the trials on which participants experienced the most difficulty, removing those trials means you are potentially removing the trials that would have increased the overall average RT for the different-response condition, rendering them significantly longer than those to non-response trials and hence revealing the expected response set effect. Nevertheless, this would not have altered the RTs to non-response set trials. If anything the RTs to non-response set trials are lower than they would have been had the more difficult trials been included. In sum, the results from Hasshim and Parris permit us to conclude that the finding of shorter RTs to same-response trials than to non-response set trials is best interpreted as supporting the notion that same-response trials involve some form of facilitation.

Whilst the present results are incompatible with multi-stage models of Stroop interference (Klein, 1964; Zhang and Kornblum, 1998; Zhang et al., 1999; De Houwer, 2003; Schmidt and Cheesman, 2005), some such models would predict that no difference should be expected between same-response and neutral trials when participants respond manually because manual responses (with color patches) do not have access to semantics (Glaser and Glaser, 1989; Sugg and McDonald, 1994; Sharma and McKenna, 1998). Given the use of a manual response with color patches in the present study our data are compatible with such models. However, it is clearly not possible to have same-response trials when using a vocal response, thus we restrict our interpretation to models whose predictions are not modified by response modality.

In the present study we also controlled for response contingency effects to ensure that such effects were not contributing to the RTs on congruent and same-response trials. One surprising effect of controlling for response contingency was the lack of Stroop facilitation effects (neutral-congruent RTs) when we had observed Stroop facilitation when contingency was not controlled in Experiment 1. The mechanism behind Stroop facilitation effects is debated (MacLeod and MacDonald, 2000; Kane and Engle, 2003; Roelofs, 2010; Brown, 2011). Our study was not designed to make this comparison, but we are not aware of any other study that has made a comparison between neutral and congruent trials when contingency is, and is not, controlled. A future study designed explicitly to test for effects of contingency would benefit from a within-subjects comparison to investigate whether, once contingency is controlled, the resulting increase in RTs to congruent trials leaves no facilitation effects to be explained.

A further effect of controlling for contingency is that, in the present data set at least, there was no difference between same-response and congruent trials suggesting that any difference between these two trial types is largely driven by response contingency and not semantic category conflict. More could be made of this result had previous studies not observed a semantic category conflict effect even after controlling for contingency (De Houwer, 2003; Schmidt and Cheesman, 2005). The present result then could be interpreted as showing no effect of semantic category conflict due to unusually fast responses on same-response trials; that is there is no difference between same-response and congruent trials (and neutral trials) because for whatever reason, semantic category conflict was absent from Experiment 2 of the present study. However, it is not clear why semantic category conflict would be absent in Experiment 2 but not Experiment 1. Furthermore, the RTs to same-response trials in Experiment 1 and 2 are identical (∼600 ms). Controlling for contingency was predicted to increase RTs to congruent trials and indeed RTs to congruent trials increased by ∼30 ms when contingency was controlled. In short, despite contrasting with previous results showing an effect of semantic category conflict when contingency is controlled, the null difference between congruent and same-response trials is most likely an outcome of an increase in RTs to congruent trials brought about by contingency. Notably, congruent trial RTs are also not different from neutral trial RTs which in turn are not different from same-response trial RTs. With the predicted effect of contingency and a neutral word baseline that does not involve semantic or response conflict the results are best interpreted as showing that RTs to same-response trials cannot be used reliably to determine the presence or absence of semantic category conflict. All future studies should include a neutral non-color word baseline when utilizing the 2-to-1 mapping paradigm.

Since we had removed the effects of response contingency from Experiment 2 we can be confident that the difference observed between the same-response and different-response trials is not overestimated. Indeed, a raw effect size of roughly 30 ms seems to be a common magnitude of difference between these two trial types whether contingency is controlled or not. However, as mentioned earlier the utility of same-response trials in such a comparison is questioned by the present results given they are not reliably different from neutral trials. In essence, our results suggest that the difference between different-response and same-response trials in terms of RTs is the same as the difference between different-response and neutral trials, meaning that it is a measure of Stroop interference and not a purer measure of response conflict as has previously been assumed (De Houwer, 2003; Schmidt and Cheesman, 2005; van Veen and Carter, 2005; Steinhauser and Hubner, 2009). The analyses on error rates also do not clearly explicate the differences between the different conditions although the trend does suggest a higher error rate for different-response trials generally, which is to be expected. Previous studies using the Stroop task typically do not focus on error rates because the relatively easy task keeps speed-accuracy trade-off to a minimum. Thus the analyses on RTs are the main focus of this paper as well.

The sample size of the present study was selected to match that of Schmidt and Cheesman (2005). However, Schmidt and Cheesman do not report the gender of their participants and so it was not possible to establish whether our participants differed from theirs in that respect. Whilst unlikely it is possible that the differences between our study and theirs (i.e., the effect of contingency on the difference between same-response and congruent trials) were a consequence of the gender differences in the present study. However, we have no reason to assume that gender would influence the present results. Nevertheless, future studies should consider testing equal numbers of male and female participants to eliminate this as a possible account of findings observed.

In conclusion, same-response trials cannot be used to determine the presence or absence of semantic category conflict, at least until the mechanisms contributing to RTs are better understood. Nor can they be used to index a purer measure of response conflict. Notably, the lack of difference between same-response and neutral trials does not necessarily mean that the two trial types are processed in a similar way. For example, van Veen and Carter (2005) have shown that different brain regions are activated by same-response and different-response trials when both are compared to congruent trials. Whilst our data suggest that any differences observed in previous studies between same-response and congruent trials is likely just greater semantic/response facilitation effects on the latter, it is possible that the competing influences of response facilitation and semantic conflict interact to influence response latency. Sometimes one might win over the other, producing evidence for conflict or facilitation, but until it is known how latency is modulated by each, or even that it actually occurs, RTs to same-response trials must be interpreted with caution. The inability to differentiate neutral and same-response trials is important and reason enough to doubt the latters usefulness in measuring semantic category conflict. Our results show that non-response set trials are potentially a better alternative.

## Conflict of Interest Statement

The authors declare that the research was conducted in the absence of any commercial or financial relationships that could be construed as a potential conflict of interest.

## Footnotes

1 To calculate a Bayes factor one must first consider the expected magnitude of the effect under investigation. Schmidt and Cheesman (2005) used experimental methods that most closely resemble the present study and observed a semantic category conflict effect of 24 ms when using congruent trials as the baseline. The size of this effect is comparable, but is at the lower end of the 24–45 ms range observed in other studies using two-to-one mapping in Stroop task (However, the larger value was in a study that presented word primes prior to the Stroop stimuli which may have encouraged greater word processing and thus greater facilitation (Parris et al., 2013). The remaining values range between 15 and 27 ms. If 15 ms, then of the 31.6 ms average raw effect size for the same-response vs. congruent trial comparison we might expect 15 ms to be facilitation (neutral-congruent) and 16.6 ms semantic category interference; in other words the RT for neutral trials falls roughly half-way between congruent and same-response trials. If 27 ms then we might expect only 3 ms interference. We would certainly not expect the difference between same-response and neutral trials to be greater than the 45 ms maximal difference observed between same-response and congruent trials so we set 45 ms as the upper bound of expected range. To set the lower bound we must consider the smallest raw effect size that would be theoretically interesting. Notably harder to define we selected 6 ms since this is the raw effect size of a recent study using the Stroop task that was theoretically meaningful (Risko et al., 2006).
